# Close of the Volume

**Published:** 1885-12

**Authors:** 


### CLOSE OF THE VOLUME.

This number is the last for the year 1885. The record of the volume is one of which we believe that the publishers have no need to feel ashamed. We have presented to our subscribers nearly seven hundred pages of sound professional reading matter. We have published about one hundred original articles, some of them of great merit. We have given full reports of all the most important society meetings. We have kept the readers of the journal well informed concerning the current professional events of the year. We have not refrained from free and full comment upon whatever affected the interests of the profession. We have, in the book notices, kept the readers posted in the literature of the profession. In short, we honestly believe that Volume VI of the INDEPENDENT PRACTITIONER presents a fair epitome of all that has been said and done in dentistry during the year 1885.

And now, what about 1886? With added knowledge and experience, and with increased facilities, we think that Volume VII will greatly exceed Volume VI in interest and value. But to succeed we must have the co-operation of the profession. There is a place for an independent journal among us, and dentists owe a duty to their literature which can only be paid with their subscriptions. We have no manufacturing or mercantile house to sustain us. We have no traveling salesmen to secure for us a clientele, or to carry to dentists the knowledge of what we offer them. We must depend solely upon the merits of the journal to secure support, and upon the good words of its admirers to get new subscribers. Every dollar that is paid to the journal is expended upon it, for our only aim is to do something for a profession that we love. We hope to lose not one of our present subscribers. May we not also expect that they will show their appreciation of our labor by securing for it new patrons? It is *your* journal. Will you not give it your active support? Send in your subscription at once, and while you are about it ask your most intimate professional friend if he will not take it too. Whenever any subscriber sends in another name the deed is not forgotten, but is marked and fully appreciated. Many of you have expressed admiration for the journal; will you not give that approval a practical form by securing for us one new subscriber?

The publishers have determined that, with the commencement of the year, some changes shall be made that will give us more room for items of professional news. There will also be one change in the publishing board. There is an immense amount of labor necessary for the proper conduct of such a journal as this, and Dr. Wm. Jarvie finds his time so fully occupied with professional and business cares, that he is unable to devote to it the attention which it demands. He has, therefore, with the consent of his associates, transferred his interest to Dr. Albion M. Dudley, of Salem, Mass. We sincerely regret the loss of the prestige of the name of Dr. Jarvie, but we are consoled by the certain knowledge that his sympathy and active co-operation, so far as his other engagements will permit, will still be ours.

Dr. Dudley is well known to the profession as a concise and graceful writer, an indefatigable worker, and one whose sympathies are deeply engaged in everything that affects the welfare of his profession. He will give special attention to the interests of the INDEPENDENT PRACTITIONER in New England, and we bespeak for him the full consideration and co-operation of all the dentists in that section, who have any regard for their professional literature.

